# Age-related fragmentation of the motor endplate is not associated with impaired neuromuscular transmission in the mouse diaphragm

**DOI:** 10.1038/srep24849

**Published:** 2016-04-20

**Authors:** Silvia Willadt, Mark Nash, Clarke R. Slater

**Affiliations:** 1 Novartis Institutes for Biomedical Research, Basel, Switzerland; 2 Institute of Neuroscience, Newcastle University, Newcastle upon Tyne, United Kingdom

## Abstract

As mammals age, their neuromuscular junctions (NMJs) gradually change their form, acquiring an increasingly fragmented appearance consisting of numerous isolated regions of synaptic differentiation. It has been suggested that this remodelling is associated with impairment of neuromuscular transmission, and that this contributes to age-related muscle weakness in mammals, including humans. The underlying hypothesis, that increasing NMJ fragmentation is associated with impaired transmission, has never been directly tested. Here, by comparing the structure and function of individual NMJs, we show that neuromuscular transmission at the most highly fragmented NMJs in the diaphragms of old (26–28 months) mice is, if anything, stronger than in middle-aged (12–14 months) mice. We suggest that NMJ fragmentation *per se* is not a reliable indicator of impaired neuromuscular transmission.

At the neuromuscular junction (NMJ), motor nerve impulses arising in the central nervous system are transmitted to skeletal muscle fibers, triggering their contraction^1^,^2^. It has been known for many years that as mammals, including humans, age, the structure of their NMJs change. This was first detected as an increase in the extent and complexity of the branches of the motor nerve terminal that make contact with the muscle fiber^3^,^4^,^5^. This increased branching was subsequently shown to be paralleled by ‘fragmentation’ of the region of synaptic differentiation of the adjacent muscle fiber surface, usually assessed from the distribution of labelling of the receptors for the transmitter, acetylcholine (AChR)^6^. Since it is also well-known that mammalian muscles become weaker with age^7^,^8^,^9^, it was natural to ask whether these changes in NMJ structure had any impact on the efficacy of neuromuscular transmission that might lead to impaired muscle activation.

Based on extensive data from humans, obtained by ‘single fiber electromyography’ (SFEMG), there is evidence of a modest impairment of neuromuscular transmission in some, but not all, muscles with age^10^,^11^. However, the increased temporal dispersion (‘jitter’) revealed by this method (on average less than 5 μs greater at 80 years of age than at 30 years of age) is much less than the increase of 80–100 μs which causes ‘blocking’ of neuromuscular transmission and would therefore lead to muscle weakness^12^. Electrophysiological studies of isolated nerve-muscle preparations from rats and mice has shown that, if anything, there is an enhancement of transmission during the first 2–2.5 years of life, apparently resulting from an increase in the number of transmitter quanta released from the nerve terminals by individual motor nerve impulses (the ‘quantal content’, QC)^13^,^14^,^15^,^16^. However, these studies relied on comparisons of populations of NMJs rather than correlations of the structure and function of individual NMJs. This left open the possibility that impairment of transmission at small, but potentially significant, numbers of highly fragmented NMJs might have been missed.

In this study, we have assessed neuromuscular transmission at mouse NMJs whose structure was determined from fluorescent labelling of the AChRs with trace amounts of fluorescent-tagged α-bungarotoxin (R-α-BgTx). This has allowed us to test quantitatively how key features of transmission are related to the number of fragments of differentiated postsynaptic membrane. We have compared NMJs from mice of 2 ages, 12–14 months and 24–28 months. These are similar to the ages studied by a number of other authors as representing ‘adult’ and ‘aged’ rats and mice^6^,^13^,^17^,^18^. In particular, an earlier study has shown that the number of synaptic regions at mouse NMJs, defined by labelling the nerve terminal, remained constant during the first year of life and then steadily increased over the next 1–2 years^6^. We thus took our observations from 1 year-old mice as a reference against which to compare values from the older mice.

Our findings confirm those from earlier comparisons of the structure and function of populations of NMJs: we find no decline, and even some evidence of an enhancement, of key features of neuromuscular transmission with increasing NMJ fragmentation. We thus conclude that NMJ fragmentation *per se* does not imply a decline in fundamental features of transmission.

## Results

### Effect of age on the structure of NMJs in mouse diaphragm

To study NMJ structure, we labelled the acetylcholine receptors (AChRs) in isolated mouse phrenic nerve–diaphragm preparations with trace concentrations of fluorescent α-bungarotoxin (see Methods).

**Figure 1:**
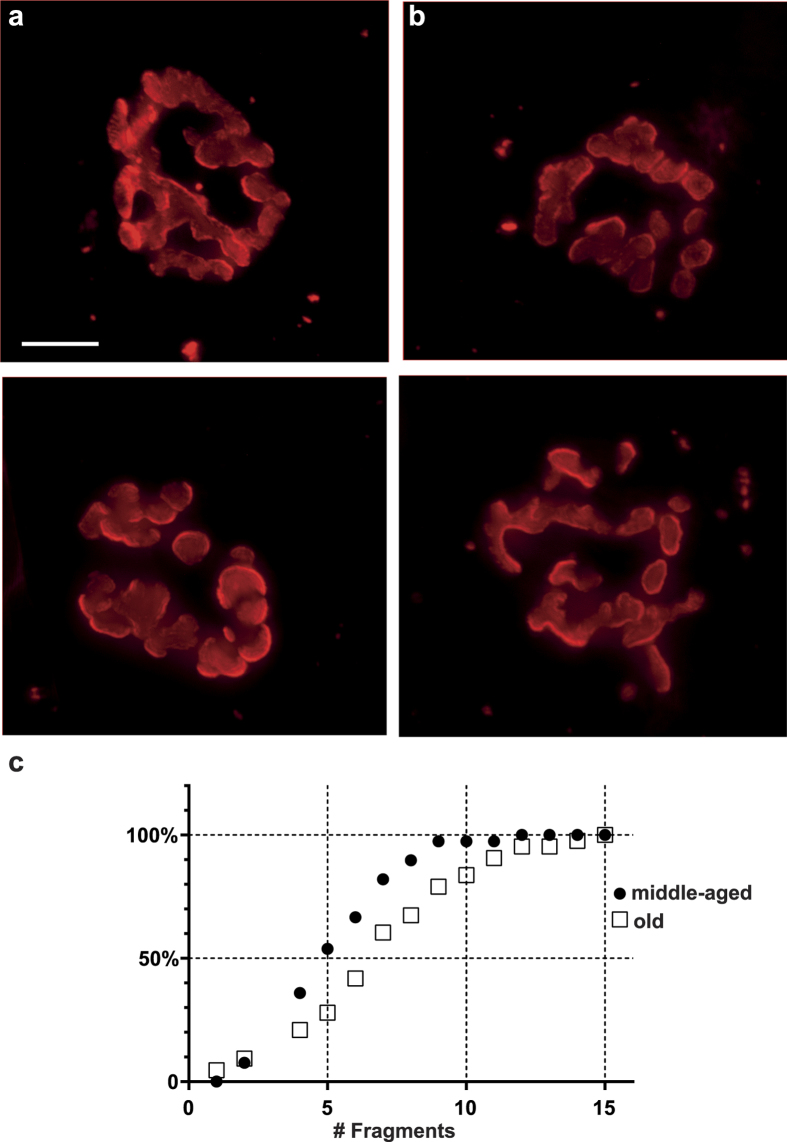
Appearance of NMJs in middle-aged and old mice. (**a**) Images of middle-aged NMJs (top: 6 fragments, bottom: 7 fragments). (**b**) Images of old NMJs (top: 11 fragments, bottom: 10 fragments). Scale bar = 12.5 μm. (**c**) cumulative histograms of the number of fragments present at NMJs in middle-aged mice (solid symbols) and old mice (open symbols).

There was no obvious qualitative difference between the appearance of the NMJs from middle-aged and old mice (Fig. 1a,b). As a result, during subsequent ‘blind’ analysis of NMJ structure, it was not possible to tell which NMJs were from old mice and which were from middle-aged ones (see Methods). Nonetheless, analysis of the number of fragments of AChR-rich membrane at individual NMJs revealed a significant increase of about 27% in the average number of fragments in the old animals (Table 1). This corresponded to an average increase of about 1.5 fragments at each NMJ (from 5.6 to 7.1). Particularly noticeable was the appearance in the old mice of a substantial number of NMJs with 10 or more fragments (Fig. 1c). These represented only 2.6% (1/38) of NMJs in middle-aged mice but 22% (9/41) in old mice. We conclude that a significant increase in the number of fragments must have occurred at these NMJs after 1 year of age.

In spite of the increase in the number of fragments, there was no significant change in either the total area occupied by those fragments (228.1 vs 235.9 μm^2^, middle-aged vs old) or the ratio of the overall length to width of the NMJs (1.43 vs 1.53) (Table 1). In our sample, there was also no difference in muscle fiber diameter between middle-aged and old muscles (Table 1).

### Effect of age on NMJ function

*cMAPs.* To assess the overall efficacy of neuromuscular transmission, we recorded compound muscle action potentials (cMAPs) from the isolated muscles, using extracellular electrodes, in response to trains of 25 maximal stimuli to the nerve at 5 Hz. This frequency is similar to that used in clinical tests for impaired neuromuscular transmission^19^ in which a decline of cMAP amplitude (“decrement”) indicates a failure of transmission at same NMJs. It allows the declining amplitude of the cMAP to be assessed in the absence of the facilitation that occurs at higher frequencies.

There was no significant change in cMAP amplitude throughout trains at 5 Hz in muscles of either age group (±2% change in the middle-aged, N = 3; ± 3% in the old, N = 11). At higher frequencies (10, 20 Hz), although no obvious consistent differences were observed, the vigorous contraction of all the muscles altered the distance between muscle and electrode, making quantitative comparisons impossible.

### Synaptic currents

The properties of neuromuscular transmission at individual NMJs were studied using a two-electrode voltage clamp to record synaptic currents in muscles in which the muscle fiber action potentials had been blocked by μ-conotoxin GIIIB (μCTX, 2–3 μM) to prevent contraction. This method allows both the amplitude and time course of the endplate currents (EPCs) to be measured. It also allows an assessment of the number of transmitter quanta released by each motor nerve impulse, the ‘quantal content’ (QC) of the response, to be made without the need for the ‘correction’ of recorded values that is required with voltage recording^20^. Although the muscles were also exposed to α-BgTx, the concentration used (10 nM for 30–40 min) caused no significant diminution of mEPP amplitude (~1 mV, Table 1) compared to previously reported values^13^.

**Figure 2:**
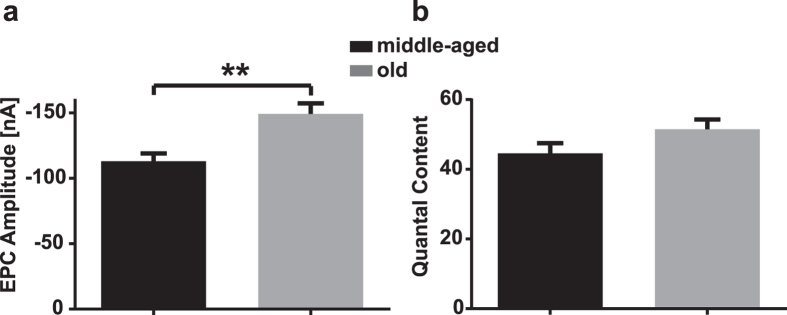
Evoked release at diaphragm NMJs in middle-aged and old mice. (**a**) Comparison of mean EPC Amplitude between middle-aged and old animals; P = 0.001. (**b**) Comparison of mean quantal content between middle-aged and old animals. P > 0.05.Values show mean ± SEM.

There was no significant difference between middle-aged and old mice in the amplitude or frequency of the spontaneous miniature endplate potentials/currents (mEPPs/mEPCs) which result from the action of individual transmitter quanta on the muscle fiber membrane (Table 1). However, the amplitudes of the EPCs evoked by nerve stimulation at 1 Hz were significantly larger in the old mice (Fig. 2a, Table 1) (−113.1 nA in middle-aged vs −149.2 nA in old mice, p = 0.001), confirming previous findings cited above. In our sample, although the average QC was about 15% bigger in the old mice than in the middle-aged ones, this difference was not statistically significant (p = 0.09) (Fig. 2b, Table 1). Neither was there any significant difference in the QC/area (0.20 quanta/μm^2^ in middle-aged vs 0.22 in old mice, p = 0.25) as might be expected if the nerve terminal in old mice occupied a smaller fraction of the postsynaptic area then in middle-aged mice.

During repetitive stimulation, there is a tendency for the evoked responses (EPPs or EPCs) to decline in a frequency-dependent manner (often referred to as ‘run-down’) during the first 20–25 responses in a train, at which point a plateau value is reached^21^. Even at 1 Hz, as used in this study, the ratio of the average amplitude of the 21st–25^th^ EPCs to the amplitude of the initial EPC in the train was about 0.75 in the middle-aged mice. In the older mice, however, there was significantly less run-down (12 mo: run-down 0.756, SD 0.130, N = 36. 28 mo: run-down 0.834, SD 0.103, N 41, p = 0.0046).

### Correlation of structure and function at the level of individual NMJs

**Figure 3:**
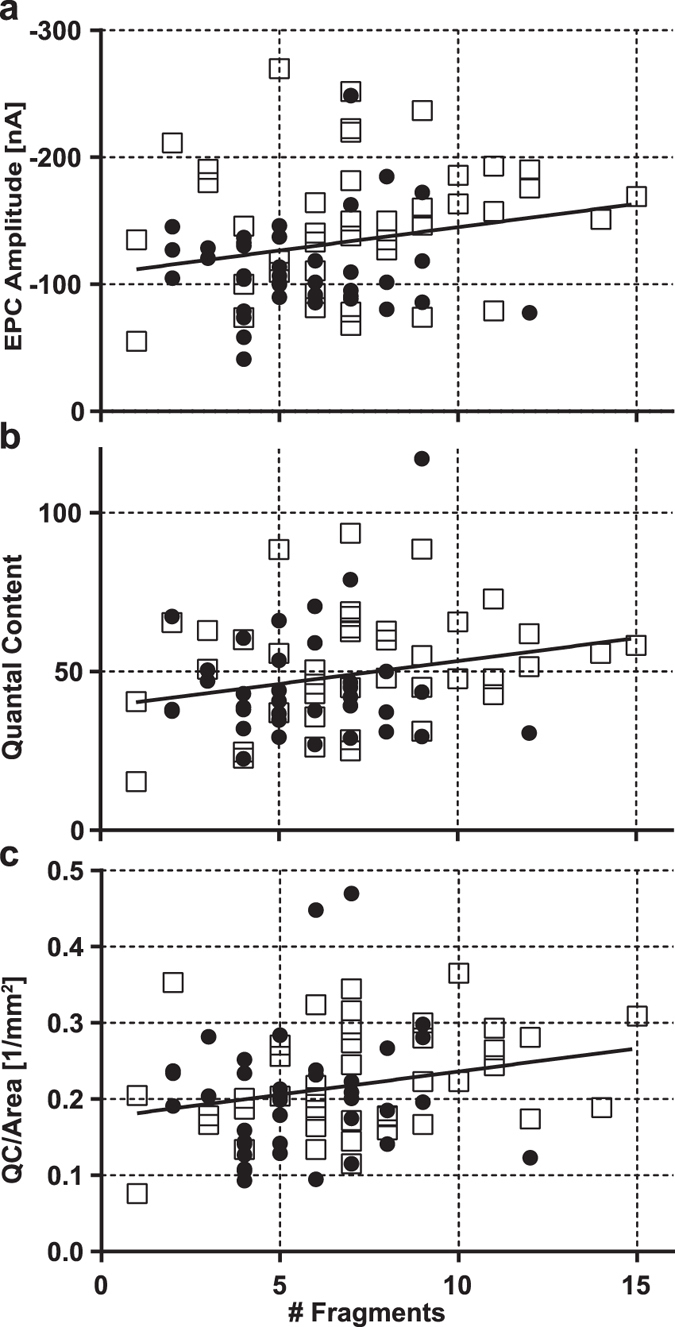
Evoked release at mouse diaphragm NMJs containing different numbers of fragments. Solid symbols, middle-aged mice; open symbols, old mice. The slope of the least-squares trend line does not differ significantly from zero for any of the three measures of release (in all cases n = 82, correl. coeff. < 0.19, p ≥ 0.1).

To test the hypothesis that increased NMJ fragmentation *per se* is associated with a decline in the efficacy of neuromuscular transmission, we looked to see whether key measures of neuromuscular transmission at individual NMJs (EPC amplitude, QC, QC/μm^2^), independent of the age of the animal, were significantly correlated with the number of fragments. In each case there was a positive correlation, implying enhanced (rather than impaired) transmission with age, but in no case was the correlation statistically significant (Fig. 3).

As noted above, a substantial fraction of the NMJs from the old, but not the younger, mice had ≥10 fragments. These NMJs were of particular interest since they represent individual NMJs which had become increasingly fragmented after 12 months of life. Inspection of Fig. 3 shows that there is no suggestion of a decline in transmission at these NMJs. Comparison of the average values of the 3 variables illustrated in Fig. 3 for NMJs with ≥10 fragments with the all the NMJs studied, or with those with <10 fragments, whether pooled, or in old or middle-aged mice treated separately, revealed no statistically significant differences. However, when the effect of repetitive stimulation on the amplitude of the EPC was compared in the two groups, it was found to be significantly less at the more fragmented NMJs (1–9 fragments; run-down 0.777, SD 0.127, N 68. ≥ 10 fragments; run-down 0.879, SD, 0.067, N 10, p = 0.001).

### Comparison of NMJs in diaphragm and EDL

There is evidence that fast-twitch fibres are particularly prone to age-related atrophy^22^,^23^,^24^. Extensor digitorum longus (EDL) muscles contain a higher proportion of fast-twitch fibres (Types IIx and IIb) than many other muscles in the mouse e.g. 90% vs 40% in diaphragm and 10% in soleus^25^. We therefore investigated a small number of EDL NMJs, in both middle-aged and old mice, to see if they differed in any obvious ways from NMJs in the diaphragm.

When compared to NMJs in the diaphragm of 12 month-old mice, those in EDL have a significantly greater overall synaptic area (EDL, 322.2; diaphragm, 228.1 μm^2^) and L/W ratio (EDL, 2.92; diaphragm, 1.43), but contain fewer fragments (EDL, 2.83; diaphragm, 5.58) (Table 2). The mEPCs are slightly smaller than in diaphragm (EDL, 2.42; diaphragm, 2.65 nA), but the EPCs are considerably larger (EDL, 252.8; diaphragm, 113.1 nA), and the QC is more than twice that in diaphragm (EDL, 105.6; diaphragm, 44.6).

None of these properties of EDL NMJs changed significantly during the second year of life (Table 2). Thus, although our sample is small, these results show that the NMJs in EDL differ consistently from those in diaphragm, and that these differences are maintained during the second year of life. They provide no evidence of a general change in the function of individual NMJs with age.

## Discussion

Our study is the first we are aware of to test directly the assumption that the progressive ‘fragmentation’ of individual NMJs, such as occurs naturally with age, is correlated with a decline of the efficacy of neuromuscular transmission at those NMJs. We found no such decline. This conclusion is based two-electrode voltage clamp studies, in conditions which allow comparison of the structure and function of individual NMJs, and is supported by cMAP recordings during stimulation at moderate frequencies, which reflect the summed behaviour of many NMJs.

Our observations of the effect of age on the structure of NMJs are broadly in line with earlier work. We found a significant age-related increase in the number of fragments into which the AChR-rich postsynaptic membrane is divided. This confirms numerous previous studies on mammalian NMJs which have found progressive age-related changes of the pattern of branching of the nerve terminal and/or the conformation of the differentiated postsynaptic membrane associated with it 3,4,5,17,26,27,28,29,30,31. For example, in a study of NMJs in a variety of muscles in C57Bl6 mice, the same strain we used, 81% of NMJs in diaphragms of mice 22–28 months old contained 5 or more fragments^26^. In our study the comparable figure is 79% (Fig. 1c).

While criteria such as ‘5 or more’ fragments have sometimes been used to classify NMJs as being either ‘fragmented’ or ‘non-fragmented’, our findings show that the number of postsynaptic fragments at individual NMJs varies widely within muscles of both age groups, and that there is extensive overlap of the distributions of fragment number in muscles from both middle-aged and old mice (Fig. 1b). This suggests that there is a gradual and continuous addition of new fragments during the period we have studied (12–28 months of age) rather than any abrupt transition of individual NMJs from a ‘non-fragmented’ to a ‘fragmented’ state such as occurs, for example, when individual muscle fibers regenerate after damage^32^,^33^. This view of the gradual nature of fragmentation is consistent with earlier observations that showed, in 5-month old mice, an average of 7.1 fragments/NMJ in EDL and of 6.0 in soleus muscles. These values had declined slightly by 1 year (to 6.8 and 4.4 respectively) and then increased substantially by 30–32 months (to 11.9 and 9.4 respectively). Although these data are based on staining of the nerve terminal, rather than the AChRs as in our study, they support the view that most NMJs contain multiple fragments, and that the number of these increases in later life.

We also confirmed previous studies in showing an enhancement of neuromuscular transmission with age^13^,^14^. The average EPC amplitude was 32% greater at diaphragm NMJs in our old group than in our middle-aged group. This difference is somewhat greater than the increase of 16% found using similar methods in the rat diaphragm^14^, the only other study to use current recording in conditions of unblocked release. Although we observed a 15% increase in QC with age, that difference was not statistically significant. However, we found that ‘run-down’ of the EPC, which is primarily a result of a decline in the number of quanta released^21^, was significantly less in 28 than in 12 month old mice. Thus both basal release, and the resistance to ‘run-down’, are greater in the older mice.

Several other studies of NMJ function have also compared values of QC in middle-aged and old rodents, although none used exactly the same combination of methods, species and muscle that we did. In those studies in both mouse and rat diaphragm muscles, QC estimated from voltage recordings increased ~50% between 10–12 and 28–30 months of age^13^,^15^. Our results from mouse diaphragm NMJs are consistent with those of previous studies in rodents showing both an increase in the number of postsynaptic ‘fragments’ and in the efficacy of neuromuscular transmission with age.

As in previous detailed studies of neuromuscular transmission in aging rodents^13^,^14^,^15^,^16^, ours was made in experimental conditions which differ considerably from those present *in vivo*. Most notably, the electrophysiological recordings were made from muscles maintained at room temperature (25 °C) rather than at body temperature. However, although there are no strictly comparable studies in mice, studies of rat diaphragm show that the quantal content of release, measured at 1 Hz, is quite insensitive to temperature in the range 25–37 °C^34^. A second difference concerns the frequency of stimulation. We have used 1 Hz to estimate the baseline QC which, in a number of previous studies, has been found to be roughly proportional to the NMJ area^21^. We have confirmed such a relationship in mice of both ages we studied. In future studies, it would be interesting to examine in detail the effects of stimulation at higher frequencies, and with patterns resembling those that occur *in vivo* (e.g. bursts of 5–10 impulses at up to 100 Hz)^21^, on the ability of nerve terminals with different numbers of fragments, in different muscles, and in animals of different ages, to maintain release.

The main aim of our study was to determine whether NMJ fragmentation, *per se*, was associated with a decline in the efficacy of synaptic transmission. Our evidence provides no support for such a decline. We found no significant correlation between EPC amplitude, overall synaptic area, or quantal release/area, and the number of separate postsynaptic fragments. This suggests that as NMJ remodelling takes place with increasing age, the newly formed synaptic sites that give the NMJ it’s increasingly spotty, or ‘fragmented’, appearance acquire essentially normal functional properties.

There are other examples of NMJs whose structure is abnormal at the level of the light microscope but which function well. These include the junctions formed on regenerated muscle fibers^32^,^35^,^36^, which consist of a compact cluster of distinct ‘spot’ contacts, and the highly abnormal junctions formed in mice during recovery from botulinum intoxication, particularly after repeated injections^37^,^38^,^39^,^40^, which consist of spot contacts that are often spread out along the muscle fiber.

Together with earlier studies, our observations show that age-related structural changes of the NMJ occur gradually with time and suggest that they arise as the outcome of an active process of new synapse formation. The success of this process is evidenced by the maintained, and even increased, efficacy of neuromuscular transmission at old and fragmented NMJs. An important implication of the striking ability of mammalian NMJs to maintain an effective level of neuromuscular transmission, after even very extensive remodelling, is that the overall conformation of an NMJ may not be an accurate predictor of its functional properties. What our study does not rule out is the possibility that in even older mice, withdrawal of the nerve terminal, possibly as a consequence of increasing partial denervation as motor neurons die, may lead to impaired transmission.

## Methods

### Animals

Animal experiments were performed in accordance with the Swiss ordinance on animal experimentation, after approval by Kantonales Veterinäramt Basel-Stadt (Schlachthofstrasse 55, 4056 Basel). Adult C57BL/6J male mice aged 12 and 24 months were purchased from Janvier Laboratories (France) and subsequently maintained in Novartis animal care facilities at 22 °C in a 12-h light–12-h dark cycle with unrestricted access to regular diet and water.

### Muscle preparation

12–14 month-old and 26–28 month-old C57Bl/6 mice, referred to as middle-aged and old, were sacrificed and the left hemi-diaphragm with about 3 cm of the left phrenic nerve was dissected and pinned out in a Perspex recording chamber volume 2 ml, with a Sylgard™ base. For experiments with extensor digitorum longus (EDL) muscles, the muscle and about 3–4 cm of the peroneal nerve were dissected and pinned out in the chamber.

Muscles were kept at room temperature (25 °C) in gassed (95%O_2_–5%CO_2_) bathing solution containing (mM): 12 NaHCO_3_, 1 KH_2_PO_4_, 1 MgCl_2_, 11 glucose, 4 KCl, 138.8 NaCl and 2 CaCl_2_ at pH7.4^41^. The proximal end of each nerve was drawn into a suction electrode for stimulation. Muscles were incubated with 10 nM α-bungarotoxin (α-BTX) (Alexa Fluor® 488 conjugate, Invitrogen) for 30–40 min at the beginning of the experiment to allow visualization of the acetylcholine receptors (AChR). The labelling conditions were such that the recorded mEPPs were within the range of values previously reported for mouse diaphragm (0.74–1.16 mV)^13^.

### Electrophysiology

Compound muscle action potentials (cMAPs), whose amplitude reflects the summed activity of many muscle fibers, were recorded at the beginning of many experiments to provide an overall assessment of the efficacy of neuromuscular transmission^37^. cMAPs were evoked by applying stimuli of 0.5–5 V and 0.1 ms duration to the nerve. Extracellular field recordings of cMAPs were made with glass micropipette electrodes, broken off to a tip diameter of about 100 μm and filled with bathing fluid. The electrode was positioned approximately 100 μm above the muscle in a region close to the NMJs, as determined by a minimal cMAP latency and an initial downward deflection.

Intracellular recordings of synaptic events from individual muscle fibers were made with sharp glass microelectrodes (5–10 MΩ) filled with 3 M KCl^42^. To abolish muscle action potentials, and hence muscle contraction, muscles were pre-incubated for 30 min in 2–3 μM mu-conotoxin GIIIB (μCTX) (Alomone Labs), which is a relatively specific blocker of the voltage-gated sodium channels in muscle^21^. The μCTX solution was then washed out and subsequent recordings were made in flowing (~1 ml/min) gassed bathing solution. The effect of the μCTX begins to wears off after ~1 hr, at which time it was reapplied.

Suitable NMJs for investigation were identified from the appearance of the labelled AChRs, viewed with a 40x water immersion objective (Zeiss Examiner.Z1 microscope). Only surface fibers were studied, and when possible we selected those in which most of the NMJ could be seen. Before making recordings, a focal series (1 μm separation in the z-axis) of images of the α-BTX fluorescence was recorded, as well as a brightfield image of the NMJ and its host muscle fiber. Two electrodes separated by 50–100 μm, one to record voltage and one to inject current (Digidata 1440A, Axoclamp 900A), were then inserted into the muscle fiber at either end of the NMJ.

Subsequent voltage and current recordings were made with the resting membrane potential (RMP) set to ~−75 mV by adjusting current flow. First, spontaneous events (mEPP) were recorded in current clamp mode. The current and voltage electrodes were then configured as a two-electrode voltage clamp to record spontaneous (mEPC) and currents (EPCs) evoked by stimulation at 1 Hz.

The peak amplitude of the synaptic events, the frequency of spontaneous events, and the exponential decay time constant of the ECPs (estimated from the time when the amplitude had declined to ~60% of its peak value, were determined using Clampfit software. Quantal content (QC) was defined as the ratio of the mean peak amplitude of the EPCs to that of the mEPCs.

### Image analysis

The degree of fragmentation and the overall area of the NMJs was determined from the stack of images obtained of each NMJ, usually consisting of 3–6 images, using ImageJ. All NMJs were analyzed by the same person who had no knowledge of the age of the mouse, whether the NMJ was from diaphragm or EDL, or the functional properties of the NMJ. A maximum intensity projection of each NMJ was made on which each distinct region of AChR labelling was outlined by hand. Reference to the full stack was frequently made to make this procedure as accurate as possible. From these outlines, the number of regions and their total area was determined. In addition, the maximum extents of the NMJ parallel (length, ‘L’) and perpendicular (width, ‘W’) to the long axis of the muscle fiber were recorded. The diameter of the muscle fiber was determined from the bright field image that accompanied each stack.

### Statistics

The number of fibers, N, is quoted for each set of data in the Tables. All values are expressed as the mean of N observations ± SD. Where mean values are compared, two-tailed Student’s t tests have been used and the value of P is given. The data in Table 1 are based on an average of 61 EPCs and 67 mEPCs recorded from each NMJ. These were recorded from a total of 38 NMJs from 8 mice 12 mo old and 43 NMJs from 22 mice 28 mo old.

## Additional Information

**How to cite this article**: Willadt, S. *et al*. Age-related fragmentation of the motor endplate is not associated with impaired neuromuscular transmission in the mouse diaphragm. *Sci. Rep.* **6**, 24849; doi: 10.1038/srep24849 (2016).

## Figures and Tables

**Table 1:** Features of NMJs in diaphragm muscles of middle-aged and old mice.

	**Age**	**#Frags**	**Area (μm**^**2)**^	**L/W**	**Fiber diam (μm)**	**mEPP (mV)**	**mEPP freq (Hz)**	**mEPC (nA)**	**mEPC freq (Hz)**	**EPC (nA)**	**QC**	**QC/Area (1/μm**^**2**^)
Ave	12.4	5.58	228.1	1.43	29.3	0.97	3.54	−2.65	5.58	−113.1	44.6	0.204
SD	0.68	2.23	60.0	0.35	6.00	0.28	3.82	0.65	2.23	37.5	17.8	0.084
N	39	38	38	38	38	38	38	38	38	38	38	38
Ave	27.2	7.20	235.9	1.53	29.2	1.01	5.05	−2.74	5.47	−149.2	51.5	0.224
SD	0.96	3.24	71.3	0.59	7.69	0.29	3.55	0.58	4.06	52.4	18.1	0.070
N	43	41	41	41	41	41	41	41	41	41	41	41
P		0.011	n.s.	n.s.	n.s.	n.s.	n.s.	n.s.	n.s.	0.001	n.s.	n.s.

**Table 2:** Features of NMJs in EDL muscles of mice middle-aged (~12 mo) and old (26–28 mo).

		**#Frags**	**Area (μm**^**2**^)	**L/W**	**Fiber diam (μm)**	**mEPP (mV)**	**mEPC (nA)**	**EPC (nA)**	**QC**	**QC/Area (1/μm**^**2**^)
**12 mo**	**Ave**	**2.83**	**322.22**	**2.92**	**32.10**	**0.31**	**−2.42**	**−252.8**	**105.67**	**0.243**
middle-aged	SD	1.94	84.34	1.63	9.13	0.07	0.49	158.0	65.46	0.173
	N	6	6	6	7	7	6	6	6	4
**26–28 mo**	**Ave**	**4.90**	**405.24**	**2.40**	**33.53**	**0.31**	**−2.06**	**−218.9**	**106.30**	**0.269**
old	**SD**	**3.65**	168.81	0.98	7.57	0.06	0.18	56.1	24.36	0.049
	**N**	**21**	21	21	21	9	7	6	6	6
	**P**	n.s.	n.s.	n.s.	n.s.	n.s.	n.s.	n.s.	n.s.	n.s.

(±SD = standard deviation). Data from a total of ‘N’ NMJs in 3 animals of each age.
